# Case Report: Moving Tumor-Like Foci Behind Refractory Epilepsy-Cerebral Sparganosis Successfully Treated by Surgery After Failure of Praziquantel Treatment

**DOI:** 10.3389/fneur.2022.838849

**Published:** 2022-02-10

**Authors:** Yusi Chen, Xu Chen, Huicong Kang

**Affiliations:** ^1^Department of Neurosurgery, Tongji Hospital, Tongji Medical College, Huazhong University of Science and Technology, Wuhan, China; ^2^Department of Neurology, Tongji Hospital, Tongji Medical College, Huazhong University of Science and Technology, Wuhan, China

**Keywords:** cerebral sparganosis, craniotomy, refractory epilepsy, *Spirometra mansoni*, tunnel sign

## Abstract

Cerebral sparganosis is clinically non-specific and easily misdiagnosed, exposing patients to the risk of severe brain damage and neurological dysfunction caused by actively migrating larvae. Diagnostic biomarkers from typical cases can help to establish an early diagnosis and proper treatment. We present a 25-year-old woman who suffered from 9 years of refractory epilepsy and was misdiagnosed with glioma and subjected to surgery. The postoperative pathology confirmed granuloma, and the tumor-like foci reappeared 3 months later. Along with the “tunnel sign” on MRI, cerebral sparganosis was suspected and confirmed by positive serum and cerebrospinal fluid antibodies against *Spirometra mansoni*. The patient visited us after a failure of four cycles of praziquantel treatment, recurrent seizures and hemiplegia with basal ganglia foci. Craniotomy was not carried out until the larva moved to the superficial lobe on follow-up MRIs, and pathology revealed sparganosis granuloma. The patient became seizure-free and recovered myodynamia but had long-lasting cognitive dysfunction due to severe brain damage. This case indicated the importance of tunnel signs and moving tumor-like foci on MRI as diagnostic clues of cerebral sparganosis. An early diagnosis is vitally important to avoid severe neural dysfunction by the long-living and moving larvae. Surgical removal of the larva is a critical remedy for cases failed by praziquantel treatment.

## Introduction

Cerebral sparganosis is a cerebral parasitic infection caused by the sparganum, the metacestode larva of *Spirometra mansoni*, which has a strong contraction ability, moves into the brain tissue and lives in necrotic tunnels, causing formation of a parasite granuloma, typically with eosinophil infiltration. The ovum of *S. mansoni* develops into the coracidium in the contaminated water after excreted by the definitive host (often dogs and cats) and is then absorbeds by the cyclops, its first intermediate host, in which the coracidium develops into the procercoid. The procercoid infects its second intermediate host, tadpoles, which catches the infected cyclops and develops into the sparganum in the muscle as tadpoles grow into frogs. Contact to either infected first or second intermediate hosts can cause cerebral sparganosis. The clinical manifestation is usually non-specific and depends on the lesion location, including headache, epileptic seizure, mild hemiparalysis and blurred vision. Blood and cerebral spinal fluid (CSF) antibodies against *Spirometra mansoni* have high sensitivity and specificity for diagnosis. Neuroimaging can provide diagnostic clues, such as moving lesions and typical tunnel signs, which mainly located at the border between the white and gray matter of the frontal and parietal lobes and the centrum semiovale but rarely appears in the cerebellum and the basal ganglia (1, 2). However, cerebral sparganosis is easily misdiagnosed as dysembryoplastic neuroepithelial tumor (DNET), glioma or a cerebral abscess (3) because the lesion commonly features space-occupying foci with enhancement, edema and mass effects on computed tomography (CT) or magnetic resonance imaging (MRI), and the diagnosis is challenging, especially during the early disease stage. Many cases are only diagnosed from pathological examination after surgical resection based on a presurgical diagnosis of various types of brain tumors (4). The delayed diagnosis leaves the patient suffering from severe neurological dysfunction and uncontrolled seizures, treated with polytherapy with various anti-epileptic drugs (AEDs), as in the patient we reported.

The treatment strategy includes drug therapy with praziquantel and surgical removal of the granuloma and the scolex (craniotomy as well as stereotactic aspiration) (5, 6). In Hong et al.'s study, 26 patients with sparganosis from mainland China received different therapies. Sixteen of them underwent craniotomy, seven underwent stereotactic aspiration and three were treated with praziquantel only, which had a similar effect of seizure control (7). Surgery could be an effective remedy, but it is still difficult to optimize the operation time considering the secondary damage from the surgery and the possibility of a failure to remove the granuloma (8).

Here we presented a case of cerebral sparganosis with tortuous diagnosis and treatment process because of misdiagnosis as brain tumor and subjected to surgery. Four circles of praziquantel treatment was unsuccessful though diagnosis was corrected, and the larva moved to basal ganglia with surgical contraindication. Finally, the larva was finally removed by craniotomy when it moved to the superficial part of the lobe by repeated follow-up MRI scanning. Related literature was also reviewed in this paper.

## Case Description

Our patient was a 25-year-old woman without a past medical history who complained of recurrent convulsive seizures for 9 years and a massive cerebral lesion observed 5 years prior. Her first unprovoked episode was considered an epileptic seizure at a local primary care clinic with a reported “abnormal EEG”, but no neuroimaging evaluation was performed due to her father's refusal (details unavailable), and no medication was given considering it was just one isolated episode when she was 16. A second similar episode recurred 2 years later, and she was discharged with oral valproic acid, without any other examinations due to her poor economic condition. The patient experienced tonic clonic seizures without specific aura every 3–4 months thereafter.

At the age of 21, she presented the same symptoms of poorly controlled seizures and was admitted to the local hospital, where routine magnetic resonance imaging (MRI) revealed an intracranial space-occupying lesion suspected to be a glioma. She was discharged with carbamazepine (CBZ) after her father's refusal to allow surgery. One month later, she withdrew from the CBZ due to a skin rash and was seizure free for 1 year.

**Figure 1:**
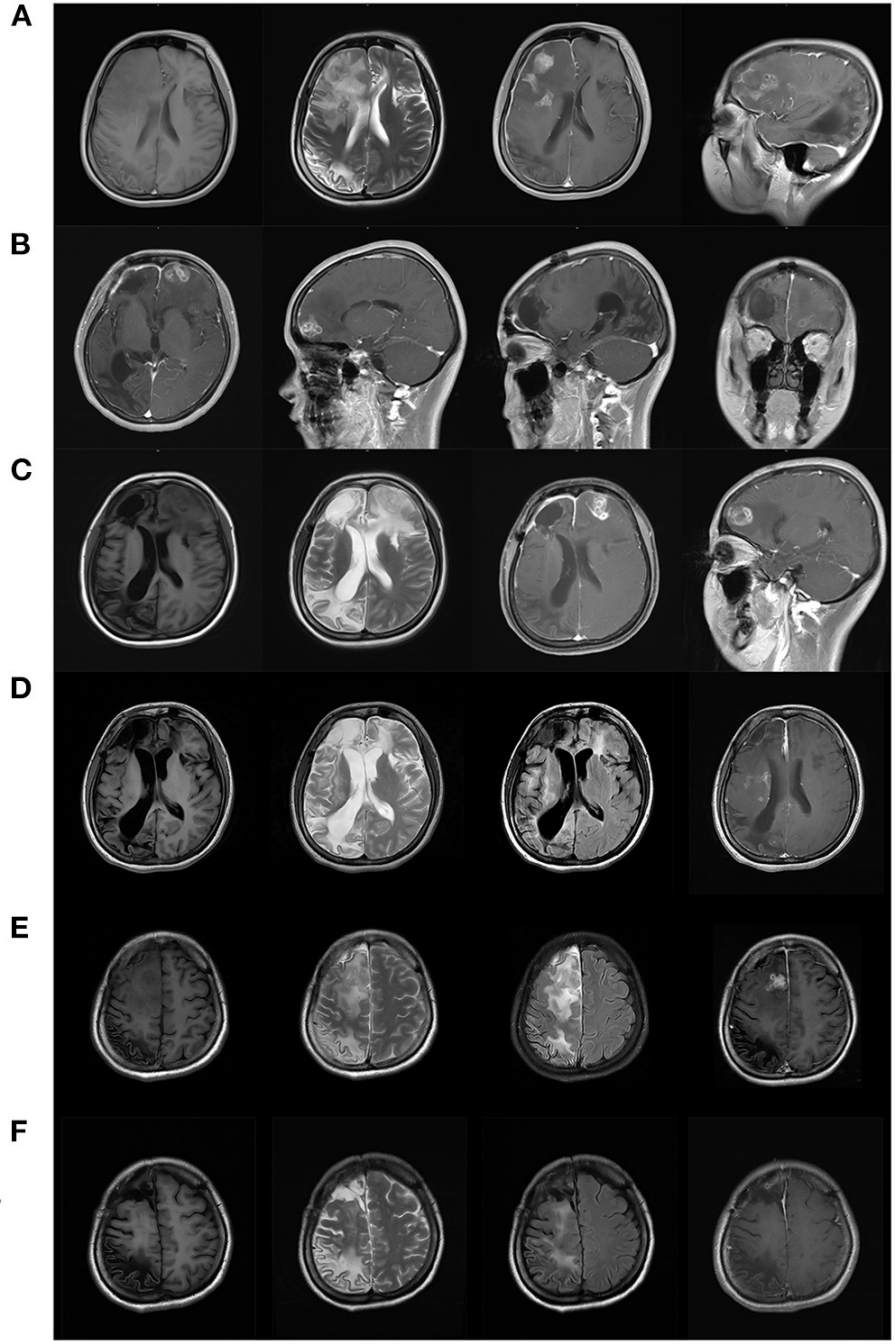
Neuroimaging results of cerebral sparganosis in the case. **(A)** Six years after the onset (aged 22), tumor-like occupation foci at the right frontal lobe with edema and irregular enhancement causing midline shift on MRI before the first operation. Note that regular ring-shaped enhancement appeared on the sagittal view (the rightmost figure) (Left to right: T1, T2, axial T1 post gad, sagittal T1 post gad). **(B)** Three months after the operation, the enhanced foci shifted to the left frontal lobe, and postoperative encephalomalacia occurred at the original right frontal lobe on her follow-up MRI images (Left to right: axial T1 post gad, sagittal T1 post gad (left side), sagittal T1 post gad (right side), coronal T1 post gad). **(C)** Five months after the operation, a typical tunnel sign at the left frontal lobe was found on both axial and sagital view of her repeated MRI with enhancement (Left to right: T1, T2, axial T1 post gad, sagittal T1 post gad). **(D)** Three years after the operation (aged 25), the patient came to our center with left side paralysis. Restricted enhancement foci in the right centrum semiovale and basal ganglia with punched-out and tunnel-like presentation were found at the right basal ganglia (Left to right: axial T1, T2, T2 FLAIR, T1 post gad). **(E)** Four years after the operation (aged 26), the larva moved to the superficial area of the right frontal lobe where surgical removal became possible (Left to right: axial T1, T2, T2 FLAIR, T1 post gad). **(F)** 16 months after the removal operation (aged 28), MRI revealed softened foci, gliosis and shrinkage of the parenchyma at the right frontal and parietal lobe. No focal or meningeal enhancement was found (Left to right: axial T1, T2, T2 FLAIR, T1 post gad).

When she was 22, she presented to the hospital again because of recurrent seizures and had a routine and enhanced MRI scan that revealed space-occupying foci with hypointensity on T1 and hyperintensity on T2 and fluid attenuated inversion recovery (FLAIR) sequences in the right frontal and temporal lobes with striped and patchy enhancement along with malacia foci without enhancement in the right frontal and parietal lobes (Figure 1A). Based on the probable diagnosis of a brain tumor, she underwent craniotomy at the sixth year after onset. However, pathology confirmed the lesion was a granuloma. The patient was discharged without AEDs and no seizure attacks after the surgery.

Three months after the operation, her follow-up MRI revealed postoperative foci with hypointensity on T1 and hyperintensity on T2 and FLAIR sequences in the right frontal lobe and encephalomalacia foci in the parietal and temporal lobes with additional tunnel-like enhancement in the left frontal lobe (Figure 1B). A detailed overview of the MRIs before and after surgery also indicated dural enhancement in the bilateral frontal lobe and anterior cingulate. It was clear that the enhanced foci had shifted to the other side and combined with her irregular parenchyma and dural enhancement and the coexistence of newer and older foci, parasitic infection was then suspected. Her history was further inquired, and her mother confirmed that she fell into a pond in their village where frogs lived at 4 years of age and underwent antibiotic treatment because of pneumonia caused by inhaling the dirty water.

Therefore, antibody tests against various parasites in her blood and CSF samples were performed, and positive results confirmed the diagnosis of cerebral sparganosis. She was prescribed anthelmintic drugs (praziquantel, details about the dosage schedule unavailable) for four cycles and levetiracetam (LEV) for seizure control. The treatment effect seemed to be satisfactory. Repeated MRI after anthelmintic treatment suggested improvement (Figure 1C), and her seizures were reduced to two to three episodes per year even after the dose of LEV was reduced to 0.5 g per day by herself.

Three years after the operation, she was 25 and suddenly developed weakness in her left extremities with a delayed response and extended sleep time. She was transferred to our epilepsy center. All physical examinations showed negative results except that the motor exam showed left-sided strength of level IV^+^ and left-sided increased tendon reflexes. Further examination confirmed decreased blood hemoglobin (106 g/L, normal range 110–150 g/L) and slightly increased cerebrospinal fluid (CSF) total protein of 45.2 mg/dL (normal range 10–45 mg/dL). Her routine blood tests, serum electrolytes and glucose levels, liver and kidney function, and nuclear cell count and electrolytes and glucose levels in CSF were all normal. Whether eosinophils presented in CSF or not were not performed because total white blood cell count in CSF was zero at that time and cell category was disabled in our instrument as the total number of nuclear cell was <50 × 10^6^/L. Her third MRI since the onset was performed and it revealed restricted enhancement focus in the right centrum semiovale, corona radiate, insula and basal ganglia with punched-out and tunnel-like presentation, and a resective operation was contraindicated due to the high risk of hemiparalysis (Figure 1D). Symptomatic treatment with LEV was prescribed with dexamethasone 10 mg iv drip daily. Standard praziquantel treatment was advised but refused by her father because of the failure of four cycles of previous treatment. The strength of her left limbs recovered 5 days later, and she was discharged with LEV.

Unfortunately, she developed another seizure episode with upward eye gaze, convulsion, frothing at the mouth and loss of consciousness for 3–4 min, and transient weakness that lasted for several hours on her left side in the following month. Her convulsive seizures stopped but the intermittent clonic seizures of her left upper limbs continued for ~1 month even though combination of three AEDs at full dosage was given. A month later, another seizure episode recurred, and follow-up MRI noted no progression with multiple enhanced foci in the right basal ganglia, insula, centrum semiovale and corona radiata, similar to the third MRI after onset. Awake electroencephalogram (EEG) displayed intermittent generalized 4–5 Hz theta waves and 2–3 Hz delta waves in the bilateral hemispheres. Sleep EEG displayed high-extremely high amplitude 11–12.5 Hz scattered sharp wave discharges and asynchronization in bilateral hemispheres. Her father refused praziquantel treatment again, and her AEDs were further adjusted to four AEDs combination at full dosage but with poor seizure control (secondary tonic-clonic seizures once per 1–2 months and clonic seizures in her left upper limb 2–3 times per month).

**Figure 2:**
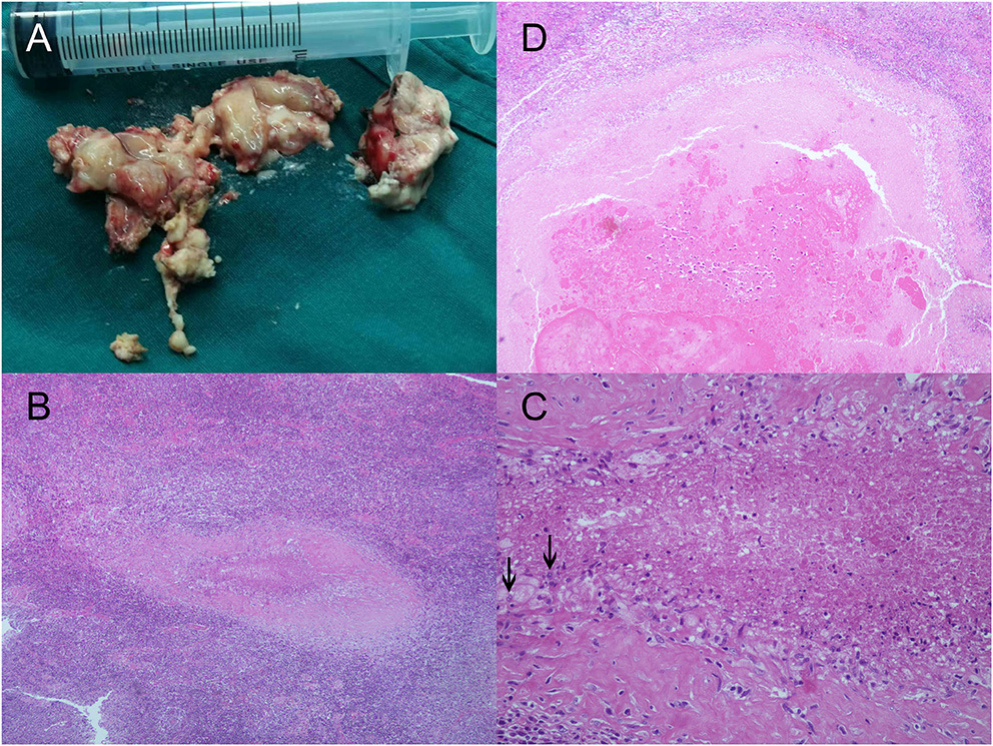
Larval samples and the pathologic results of cerebral sparganosis in the case. **(A)** Larva samples acquired during the operation in our hospital. Necrotic objects split into band shaped sections from the larva were wrapped in surrounding brain tissue. The larva adhered to the surrounding brain tissue because of severe inflammatory reactions and therefore was not fully presented. **(B,C)** Images of microscopy inspection of the sample under low power (HE, ×100) and high power (HE, ×400) are also presented, which are characterized by irregular striped necrotic objects with inflammatory infiltration of multinucleated giant cells and the formation of foreign body granulomas. Calcareous corpuscles, which were characterized by basophilic vacuolated structures in the larva body, were indicated with black arrows. **(D)** Coronal section of the necrotic object (HE, ×100).

She was admitted again to search for a better treatment strategy for her refractory epilepsy and active cerebral sparganosis. At the age of 26, her follow-up MRI revealed a new enhanced focus in her right frontal lobes, indicating that the larva had moved to the surface (Figure 1E). Her repeated blood and CSF sparganosis antibody testing still showed positive results. Considering that the effect of praziquantel therapy was limited and the larva had moved to an unimportant functional area on the surface of the frontal lobe, she was then transferred to the Department of Neurosurgery for her second operation. A parasite granuloma adhering to the surrounding cerebral tissue was found in the right frontal lobe, the same location as the enhanced areas on MRI, and was removed (Figure 2). Pathology confirmed an irregular striped necrotic object in the center of the resected tissue with inflammatory infiltration mainly by lymphocytes and plasma cells. Multinucleated giant cells and foreign body granulomas were found around the necrotic object. Blood vessels were surrounded by lymphocytes in a sleeve-like manner (Figure 2). The pathological result indicated that after four circles of anthelmintic therapy by praziquantel, the larva was dead at the end of the course and formed the parasite granuloma with inflammatory reaction and AEDs was therefore ineffective.

Her postoperative recovery was uneventful without neurological dysfunction except an increased tendon reflex of the left limbs at discharge and she was prescribed two AEDs at middle dosage. She achieved a seizure-free status in postoperative follow-up for 16 months. However, during the follow-up, the patient had impaired short-term memory and executive function, depression and daytime sleepiness and was unable to work. Her Mini-Mental State Examination (MMSE) score was 27 (losing three scores for orientation to space and time), and her Montreal Cognitive Assessment (MoCA) score was 24 (losing one score for visuospatial cognition, one score for naming, one score for attention and three scores for delayed memory). Her follow-up MRI scanning indicated only slight dural enhancement in the bilateral frontal lobe and anterior cingulate (Figure 1F), and EEG indicated intermittent middle amplitude 5–7 Hz slow waves on bilateral frontal electrodes with right side predominant. She was given escitalopram 10 mg per day along with OXC monotherapy and was still not able to return to work but was more active in daily life with an improved mood at the 18-month telephone follow-up.

Our patient was eventually diagnosed with cerebral sparganosis at the sixth year after her first seizure onset and even underwent craniotomy based on a misdiagnosis of glioma. Unfortunately, her cerebral structure and function were irreversible due to the damage caused by migration of the worm and repeated cerebral surgery. Our case demonstrates how easily cerebral sparganosis can be misdiagnosed and how important an early diagnosis and treatment is to avoid permanent and severe brain damage and to achieve a good prognosis.

## Discussion

Cerebral sparganosis is a relatively rare parasitic disease with a high misdiagnosis rate before biopsy or operation, which is up to 57.7% at the first admission according to a Chinese cohort including 52 patients (9). Cerebral sparganosis is commonly misdiagnosed as a brain tumor, brain abscess or encephalitis granuloma, mainly because of the space-occupying mass with edema and enhancement on the neuroimage (7). Cerebral sparganosis can also be misdiagnosed as transfer tumors that typically present with irregular enhancement with ring-shaped edema at a fixed position on MRI and as cerebral cysticercosis, since the patients often have a contact history of eggs of *Taenia solium* and typical cyst images in the brain (10). The current case was misdiagnosed as glioma and even underwent surgery until a shift of enhancement on MRI 3 months after surgery, indicating parasitic infection, and the antibody test confirmed *Spirometra mansoni* infection.

For diagnosis, the image presentation, immunologic tests and contact history can provide some clues. CT presents a fresh lesion and the Sparganum granuloma as a low-density edema region and nodular or stripe high-density shadow, respectively, with marked enhancement. The lesion on MRI presented as an iso- or hypointense region on T1-weighted imaging (T1WI) and an irregular hyperintense region on T2-weighted imaging (T2WI). Typical tunnel signs can be revealed as tubular or distorted bead-like structures with T1WI hypointensity, T2WI hyperintensity and evident enhancement (11). The positive result of CSF IgG antibodies against *Spirometra mansoni* is critical to the diagnosis, with high sensitivity but relatively low specificity. It is reported that among 18 patients with positive results acquired by ELISA targeting sparganosis IgG in CSF, only two was confirmed to be pathologically definite sparganosis after surgery (12). ELISA assays targeting the parasitic antigen Spirometra erinaceieuropaei cysteine protease were therefore developed to promot the specificity of serodiagnosis (13, 14). A contact history with second intermediate hosts, such as undercooked meat, frogs, snakes and birds in the carrier state or their living environment, is a predisposing factor for sparganosis. This is an important reason why sparganosis is more common in Asian countries, where eating raw snake blood or galls is popular in some areas, along with applying snake or frog blood or skin to treat wounds (15). In the current case, further inquiry when the patient came to our center reminded her of the experience of falling into a pond with copepods (first intermediate host) in her childhood. Absorption of copepods and skin/mucosa contact with copepods could cause infection, which became an important diagnostic clue.

Treatment modalities of cerebral sparganosis include the anthelmintic praziquantel and surgery removal. Although praziquantel is regarded as the standard medical treatment for human infection with trematodes and cestodes (16), conventional doses (25 mg/kg for 3 days) are frequently reported to fail in the treatment of cerebral sparganosis (17). This may attribute to the fact that the level of praziquantel in CSF is about 1/7 to 1/5 of the plasma concentration (18). It is noted that administration with a high dose but a short duration causes non-lethal injury to the parasite and could induce parasite recovery and escape. Therefore, enough treatment course should also be emphasized (19). High-dose and longer duration of treatment (75 mg/kg for 7 days) is then considered for the cerebral infection and can achieve improved effects including decreased levels of CSF antibodies, elimination of radiographical lesions, and discontinued seizures with reduced doses of AEDs (6, 8, 20). However, there still exist cases in which multiple cycles of high-dose praziquantel treatment (75 mg/kg administered in three divided doses for 10 days) failed to reduce the seizure frequency or relieve neurological deficits in more than 14% of patients after follow-up with a duration over 13 months (6). Steroids are also used to control the immune response in cerebral sparganosis and the incidence of Herxheimer reaction of praziquantel. Additionally, Although steroids were indicated to raise the permeability of blood-brain barrier and increase the concentration of praziquantel in CSF, previous studies have shown that the plasma level of praziquantel was decreased when simultaneously treatment with steroids, which may attribute to the nature of steroids as a cytochrome P450 inducer and accelerated metabolization (21, 22). Evidence for enhancing the anti-parasite efficacy of praziquantel by prescribing steroids was absent. In turn, the efficacy of praziquantel can be enhanced by co-administration with cytochrome P450 inhibitors such as cimetidine (23). Over all, we should noted that all these data were from small studies and no randomized trials existed.

Surgery for cerebral sparganosis is considered the optimal and radical treatment (24, 25), which is also confirmed in our case by the fact that the effect of previous drug treatments, including high-dose praziquantel and AEDs, was limited to symptom control (7). To guarantee the success of the operation, the scolex of the larva must be removed either by traditional craniotomy or stereotactic aspiration to avoid recurrence. Stereotactic aspiration has developed into a mature operation in the treatment of cerebral sparganosis, which causes limited wounds and prevents larval breakage, so this should be the first choice of surgery (5). With image-guided localization and aspiration from multiple directions, removal of the larva and the granuloma can be achieved. Once repeated aspiration fails, craniotomy should be considered, which could remove the larva entirely, especially for superficial and adhesive lesions caused by severe inflammatory reactions of the surrounding brain tissue. The surgery of our patient indicated that the larva was less likely to be removed by stereotactic aspiration because it was adhesive to the surroundings. This might be caused by four cycles of praziquantel treatment harming the larva, along with the long life of larvae in the brain, resulting in the focal inflammatory reaction. However, surgery is commonly contraindicated if the larva is located in functional areas, as in our case. In this case, preoperative praziquantel treatment is sometimes applied to compel the movement of larvae from functional areas, and repeated MRI will help judge the opportunity for surgery when the larva moves to be superficial. Therefore, the opportunity should be carefully considered based on the position of the larva.

## Conclusion

This report presents a case of cerebral sparganosis with long-lasting refractory epilepsy, which was first misdiagnosed as glioma and underwent craniotomy. The larva failed to be removed by four circles of praziquantel treatment but was finally removed by opportune surgery. The current case indicated the typical diagnostic biomarkers of cerebral sparganosis and confirmed that early diagnosis and complete surgical removal of sparganosis granuloma is critical to the successful treatment, control of refractory epilepsy and the avoidance of severe tissue damage by the larva.

## Data Availability Statement

The raw data supporting the conclusions of this article will be made available by the authors, without undue reservation.

## Ethics Statement

The studies involving human participants were reviewed and approved by Ethics Committee on Human Research of Tongji Hospital. The patients/participants provided their written informed consent to participate in this study. Written informed consent was obtained from the individual(s) for the publication of any potentially identifiable images or data included in this article.

## Author Contributions

YC wrote the draft of the manuscript. XC collected all the clinical data. HK gave the main idea and edited the whole manuscript. All authors contributed to the article and approved the submitted version.

## Funding

This work was supported by the National Natural Science Foundation of China (81974279), China Association Against Epilepsy Fund for Epilepsy Research-UCB Fund (2020020A), and Grants for Returned Overseas Doctors of Tongji Hospital and Health Commission of Hubei Province (WJ2021M131).

## Conflict of Interest

The authors declare that the research was conducted in the absence of any commercial or financial relationships that could be construed as a potential conflict of interest.

## Publisher's Note

All claims expressed in this article are solely those of the authors and do not necessarily represent those of their affiliated organizations, or those of the publisher, the editors and the reviewers. Any product that may be evaluated in this article, or claim that may be made by its manufacturer, is not guaranteed or endorsed by the publisher.

## References

[1] Chang KH Cho SY Chi JG Kim WS Han MC Kim CW. Cerebral sparganosis: CT characteristics. Radiology. (1987) 165:505–10. 10.1148/radiology.165.2.3659374 3659374

[2] Shirakawa K Yamasaki H Ito A Miyajima H. Cerebral sparganosis: the wandering lesion. Neurology. (2010) 74:180. 10.1212/WNL.0b013e3181c91a15 20065255

[3] Lei W Fei W. Analysis of clinical characteristics in 24 cases of cerebral sparganosis. China Trop Med. (2016) 16:698–701. 10.13604/j.cnki.46-1064/r.2016.07.19

[4] Feng C Jie W Yuqin Z. Clinical and radiological analyses of 27 cases of brain parasitic diseases. China Modern Doctor. (2014) 52:48–50.

[5] Deng L Xiong P Qian S. Diagnosis and stereotactic aspiration treatment of cerebral sparganosis: summary of 11 cases. J Neurosurg. (2011) 114:1421–5. 10.3171/2010.4.JNS1079 20486898

[6] Zhang P Zou Y Yu FX Wang Z Lv H Liu XH. Follow-up study of high-dose praziquantel therapy for cerebral sparganosis. PLoS Negl Trop Dis. (2019) 13:e0007018. 10.1371/journal.pntd.0007018 30640909 PMC6331082

[7] Hong D Xie H Zhu M Wan H Xu R Wu Y. Cerebral sparganosis in mainland Chinese patients. J Clin Neurosci. (2013) 20:1514–9. 10.1016/j.jocn.2012.12.018 23911107

[8] Hong D Xie H Wan H An N Xu C Zhang J. Efficacy comparison between long-term high-dose praziquantel and surgical therapy for cerebral sparganosis: a multicenter retrospective cohort study. PLoS Negl Trop Dis. (2018) 12:e0006918. 10.1371/journal.pntd.0006918 30346956 PMC6211769

[9] Shi DM Wang XL Chen L Xie Q. Clinical characteristics and misdiagnosis analysis of sparganosis: A retrospective study of 52 cases. J Diagn Concepts Pract. (2020) 19:37–43. 10.16150/j.1671-2870.2020.01.00919852369

[10] Abdel Razek AA Watcharakorn A Castillo M. Parasitic diseases of the central nervous system. Neuroimaging Clin N Am. (2011) 21:815–41, viii. 10.1016/j.nic.2011.07.005 22032501

[11] Song T Wang WS Zhou BR Mai WW Li ZZ Guo HC. CT and MR characteristics of cerebral sparganosis. Am J Neuroradiol. (2007) 28:1700–5. 10.3174/ajnr.A0659 17885230 PMC8134205

[12] Jin Y Kim EM Choi MH Oh MD Hong ST. Significance of serology by multi-antigen ELISA for tissue helminthiases in Korea. J Korean Med Sci. (2017) 32:1118–23. 10.3346/jkms.2017.32.7.1118 28581268 PMC5461315

[13] Rahman SM Kim JH Hong ST Choi MH. Diagnostic efficacy of a recombinant cysteine protease of *Spirometra erinacei* larvae for serodiagnosis of sparganosis. Korean J Parasitol. (2014) 52:41–6. 10.3347/kjp.2014.52.1.41 24623880 PMC3948992

[14] Liu LN Zhang X Jiang P Liu RD Zhou J He RZ. Serodiagnosis of sparganosis by ELISA using recombinant cysteine protease of *Spirometra erinaceieuropaei* spargana. Parasitol Res. (2015) 114:753–7. 10.1007/s00436-014-4270-5 25532486

[15] Wang SM Yang FF Huang YX Shi GF Weng XH. Clinical analysis of 78 cases of parasitic encephalopathy. Chin J Parasitol Parasit Dis. (2009) 27:245–8. 19852369

[16] Chai JY. Praziquantel treatment in trematode and cestode infections: an update. Infect Chemother. (2013) 45:32–43. 10.3947/ic.2013.45.1.32 24265948 PMC3780935

[17] Chai JY Yu JR Lee SH Kim SI Cho SY. Ineffectiveness of praziquantel treatment for human sparganosis (a case report). Seoul J Med. (1988) 29:397–9.

[18] Andrews P Thomas H Pohlke R Seubert J. Praziquantel. Med Res Rev. (1983) 3:147–200. 10.1002/med.2610030204 6408323

[19] Timson DJ. Praziquantel: an enigmatic, yet effective, drug. Methods Mol Biol. (2020) 2151:1–8. 10.1007/978-1-0716-0635-3_1 32451991

[20] Gonzenbach RR Kong Y Beck B Buck A Weller M Semmler A. High-dose praziquantel therapy for cerebral sparganosis. J Neurol. (2013) 260:1423–5. 10.1007/s00415-013-6901-7 23546305

[21] Vazquez ML Jung H Sotelo J. Plasma levels of praziquantel decrease when dexamethasone is given simultaneously. Neurology. (1987) 37:1561–2. 10.1212/WNL.37.9.1561 3627459

[22] Abla N Keiser J Vargas M Reimers N Haas H Spangenberg T. Evaluation of the pharmacokinetic-pharmacodynamic relationship of praziquantel in the *Schistosoma mansoni* mouse model. PLoS Negl Trop Dis. (2017) 11:e0005942. 10.1371/journal.pntd.0005942 28934207 PMC5626502

[23] Overbosch D. Neurocysticercosis. An introduction with special emphasis on new developments in pharmacotherapy. Schweiz Med Wochenschr. (1992) 122:893–8. 1615299

[24] Anders K Foley K Stern E Brown WJ. Intracranial sparganosis: an uncommon infection. Case report. J Neurosurg. (1984) 60:1282–6. 10.3171/jns.1984.60.6.1282 6726374

[25] Kim DG Paek SH Chang KH Wang KC Jung HW Kim HJ. Cerebral sparganosis: clinical manifestations, treatment, and outcome. J Neurosurg. (1996) 85:1066–71. 10.3171/jns.1996.85.6.1066 8929496

